# The role of environmental factors for the composition of microbial communities of saline lakes in the Novosibirsk region (Russia)

**DOI:** 10.1186/s12866-015-0618-y

**Published:** 2016-01-27

**Authors:** Alla V. Bryanskaya, Tatyana K. Malup, Elena V. Lazareva, Oxana P. Taran, Alexey S. Rozanov, Vadim M. Efimov, Sergey E. Peltek

**Affiliations:** Institute of Cytology and Genetics, Siberian Branch of Russian Academy of Sciences, 10 Prospekt Lavrentyeva, Novosibirsk, 630090 Russia; V.S. Sobolev Institute of Geology and Mineralogy, Siberian Branch of Russian Academy of Sciences, 3 Prospekt Akademika Koptyuga, Novosibirsk, 630090 Russia; G.K. Boreskov Institute of Catalysis, Siberian Branch of the Russian Academy of Sciences, 5 Prospekt Lavrentyeva, Novosibirsk, 630090 Russia; Novosibirsk State University, 2 Pirogova Street, Novosibirsk, 630090 Russia

**Keywords:** Microbial communities, Saline lakes, Environmental factors, Fluorescent in situ hybridization

## Abstract

**Background:**

Nothing is currently known about microbial composition of saline lakes of the Novosibirsk region and its dependence on physical-chemical parameters of waters. We studied the structure of microbial communities of saline lakes of the Novosibirsk region and the effect of physical-chemical parameters of waters on microbial communities of these lakes.

**Results:**

According to the ion content, the lakes were classified either as chloride or chloride-sulfate types. Water salinity ranges from 4.3 to 290 g L^−1^. Many diverse microbial communities were found. Filamentous and colonial Cyanobacteria of the genera *Scytonema*, *Aphanocapsa*, and/or filamentous Algae dominated in littoral communities. Spatial and temporal organization of planktonic microbial communities and the quantities of Archaea and Bacteria were investigated using fluorescent in situ hybridization. We have found that the dominant planktonic component is represented by Archaea, or, less frequently, by Bacteria. Various phylogenetic groups (Bacteria, Archaea, Algae, and Cyanobacteria) are nonuniformly distributed. The principal component analysis was used to detect environmental factors that affect microorganism abundance. We found the principal components responsible for 71.1 % of the observed variation. It was demonstrated that two-block partial least squares was a better method than principal component analysis for analysis of the data. We observed general relationships between microbial abundance and water salinity.

**Conclusions:**

We have performed the first-ever study of the structure of the microbial communities of eleven saline lakes in the Novosibirsk region along with their physical-chemical parameters of waters. Our study demonstrates that saline lakes in the Novosibirsk region contain a unique microbial communities that may become a prolific source of microorganisms for fundamental and applied studies in various fields of ecology, microbiology, geochemistry, and biotechnology, and deserve further metagenomic investigation.

## Background

Studies of natural microbial communities have long been impeded by the fact that only a small fraction of microorganisms could be cultured on selective media. Fluorescent in situ hybridization (FISH) allows one to visualize microorganisms and infer their spatio-temporal interactions directly in the native sample without having to employ cultivation, which strongly underestimates the diversity [1–3]. Over the last decades FISH has been used to study microbial communities of oceanic plankton and benthos, coral reefs and deep-sea vents, wastewater, activated sludge, and freshwater sediments [4–10]. However, in Russia, only a few microbial communities of some large freshwater and brackish water reservoirs (lakes Baikal, Shira, and Shunet, delta of the Selenga river, the Black sea) have been studied using this method [11–13].

The small territory of the Kulunda and Baraba steppes in southern West Siberia abounds in shallow saline lakes, which are commonly closed basins. The area is a hilly terrain, and this topography spreads as far as the present Arctic seas being produced either by wind erosion [14] or by tectonic uplift [15].

**Fig. 1:**
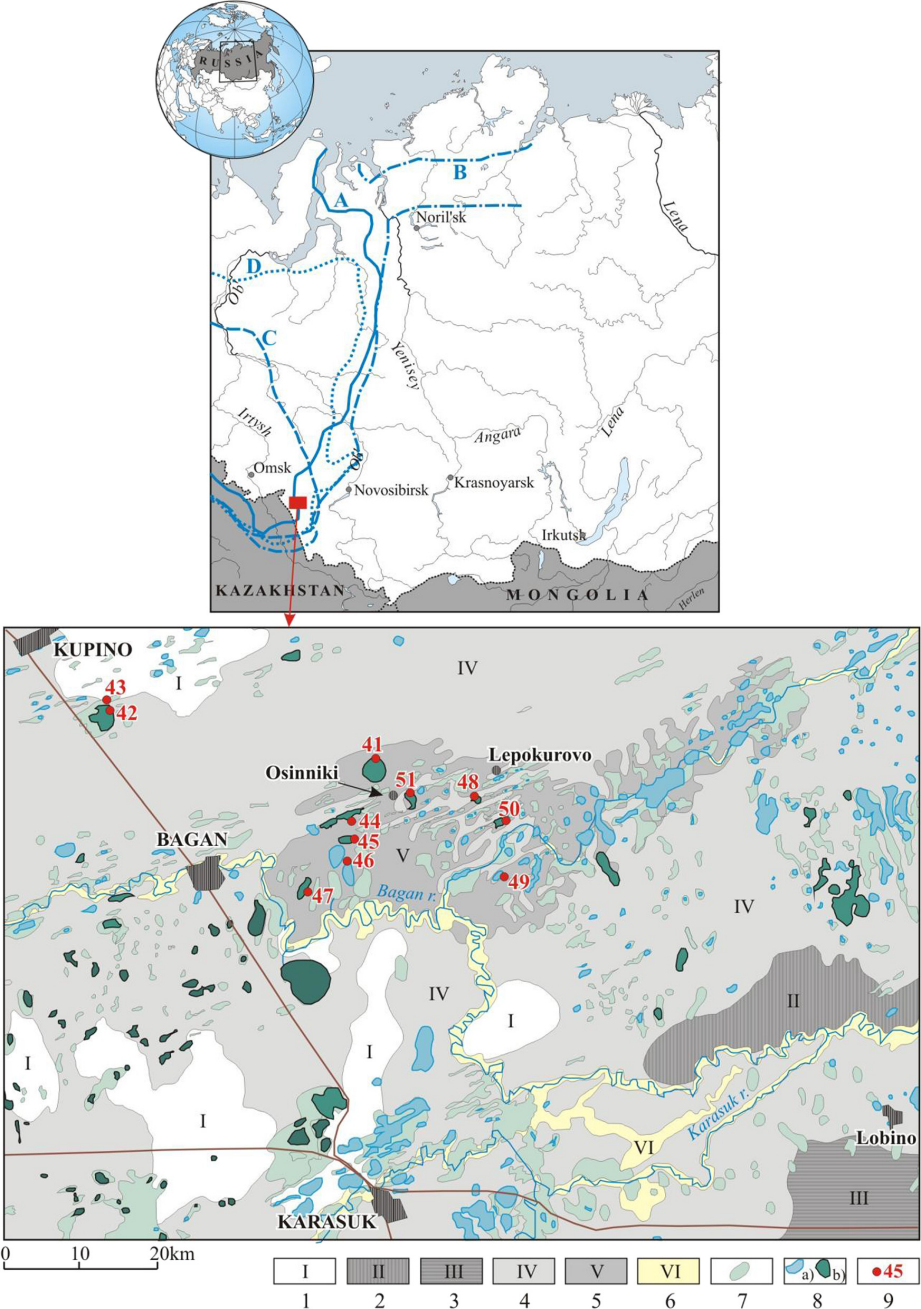
Location map of saline lakes. Blue lines in the top panel show reconstructed limits of the West Siberian sea in different stages of the Palaeogene [18]: Latest Selandian-early Thanetian (**a**), Late Thanetian–Ypresian (**b**), Lutetian (**c**), and Bartonian-Priabonian (**d**). The panel below shows simplified geology. 1 Pre-Quaternary clay silt, clay, and loess, including those building the hills; 2,3 Lower-Middle Pleistocene lacustrine-fluvial clay silt, or less often silt and sand (2) and loess and lake sand, silt, and clay silt (3); 4 Middle-Upper Pleistocene lacustrine and fluvial sand, silt, clay silt, often muddy; 5–7 Upper Pleistocene-Holocene lacustrine clay silt (often muddy), silt, and sand (5), alluvium and slope wash (6), and lacustrine-bog peat, muddy clay silt, and sapropel (7); 8 Freshwater (a) and saline (b) lakes; 9 Sampling sites

In the geological past, the territory was covered by the West Siberian inland sea extending in the N—S direction from present Kazakhstan to the Arctic basin (Fig. 1). It formed in the early Selandian (about 60 Ma according to the International Stratigraphic Chart) and repeatedly lost connection with the Arctic ocean since the Lutetian, though the water system included W—E straits linking the inland and margin seas of the Northern Perithetys and Atlantic oceans [16–18]. In Bartonian-Priabonian time, the West Siberian and Arctic basins became totally separated. The sea retreated from the West Siberian plate at the Eocene-Oligocene boundary, i.e., about 34.9 Ma. Younger rocks in the area are continental lacustrine, lacustrine-fluvial, bog, and aeolian sediments [16].

The Novosibirsk region which includes parts of Kulunda and Baraba steppes contains many saline and brackish lakes. Its climate is strongly continental. Vegetation period is characterized by a great amount of heat and sunlight. Maximum temperatures range from 40 °C to−50 °C. Annual precipitation is low, about 230–250 mm, 50–70 mm of them as snow. Warm period account for up to 70 % of precipitation [19, 20].

Most lakes in this region are very small and are characterized by short vegetation periods, high seasonal changes and waterline fluctuations, and frequent desiccations [19–21]. These lakes are highly unstable systems so microbial communities must have metabolic and behavioral adaptations to cope with the fluctuations of various environmental factors [21–24].

There are many studies of microbial communities of saline lakes of many regions [25–32]. These studies demonstrated that microbial communities of continental saline lakes are mostly represented by green Algae, Archaea, and Bacteria [25]. There are some studies on physical-chemical parameters of water, planktonic algo-and zoocenoses, aerobic chemolithoautotrophic bacteria, organic matter, and phototrophic communities of saline lakes of the Kulunda and Baraba steppes [33–38]. However, nothing is currently known about microbial composition of saline lakes of the Novosibirsk region and its dependence on geochemical parameters.

There are some data on the impact of physical-chemical parameters on the structure of microbial communities [39–45] which suggest that community composition is affected by environmental and spatial factors, such as salinity, chlorophyll *a* concentration, water color, wind, precipitation, solar radiation, temperature, conductivity, and dissolved organic carbon [39–46].

We attempted to find the key environmental factor affecting the composition of microbial communities of the Novosibirsk region. We hypothesized that salinity (concentration of main ions) is the crucial variable defining the composition of microbial communities.

In this connection, it is important to determine the composition of microbial communities of water and microbial mats of saline lakes of the Novosibirsk region, and to study the impact of geochemical parameters on these communities.

Our aim was to study the structure of microbial communities of saline lakes of the Novosibirsk region using fluorescent in situ hybridization and microscopy, as well as the effect of physical-chemical parameters of waters on microbial communities of these lakes using principal components analysis and two-block partial least squares.

The results of this study reveal the statistically significant relationships between the quantities of organisms belonging to various phylogenetic groups and physical-chemical parameters. We suggest that the structure of microbial communities of saline lakes of the Novosibirsk region is determined by physical-chemical parameters of waters, most significantly by salinity (concentration of main ions). Generally changes in salinity resulted in changes of total plankton quantity, as well as in the ratios of the main phylogenetic groups: Archaea, Bacteria, Algae, and Cyanobacteria. In the majority of cases the increase in absolute and relative quantities of Archaea was observed.

## Results

We studied 11 saline lakes located in the south of the Novosibirsk region (Fig. 1). Depth, area, and water temperature in saline lakes of the Novosibirsk region vary depending on the weather. Table 1 lists maximum depths and average areas of the lakes; water temperature was 20–25 °C when samples were taken. Bottom sediments of the studied lakes were represented by black or blue-gray silt with the smell of hydrogen sulfide, suggesting high content of organic matter. Banks were mildly sloping, covered with herbaceous vegetation. Bank edges and silt were often covered with a salt crust; rosy-brown film of *Artemia* sp. was sometimes found.

**Table 1:** Sampling locations, dates and environmental variables

Site	Lake	Coordinates	Maximum depth (m)	Area (km^2^)	Sampling frequency	Period/date
41	Gorkoye	54°14^′^N 77°58′E	4	7	24	3 Jul 08, 9 Jun 09
42	Gorkoye	54°17^′^N 77°27′E	1	10	24	4 Jul 08, 10 Jun 09
43	Presny prud	54°17^′^N 77°27′E	2	0.03	8	4 Jul 08, 10 Jun 09
44	Dolgoye	54°10^′^N 77°56′E	2	10	28	5 Jul 08, 11 Jun 09
45	Krugloye	54°08^′^N 77°56′E	1	3	24	5 Jul 08, 11 Jun 09
46	Razboynoye	54°07^′^N 77°55′E	2	4	24	6 Jul 08, 12 Jun 09
47	Khorosheye	54°05^′^N 77°51′E	1	7	28	6 Jul 08, 12 Jun 09
48	Solenoye	54°14′N 78°13′E	2	6	80	7 Jul 08, 13 Jun 09
49	Gorkoye	54°06^′^N 78°13′E	2	4	24	7 Jul 08
50	Solenoye	54°10^′^N 78°13′E	2	4	24	8 Jul 08
51	Gorkoye	54°12^′^N 77°02′E	2	4	24	8 Jul 08, 14 Jun 09

### Physical-chemical parameters of waters

It should be noted that 9 of the 11 saline lakes studied (except the lakes 43 and 46) had extremely high concentration of NaCl in the water (salinity ranged from 43 to 290 g L^−1^). In this case the task to measure the physical-chemical parameters of water becomes non-trivial. High analysis errors were due to the interfering effect of Na^+^ and Cl^−^ ions on the ion-selective electrodes as well as the multiple dilution of water for the analysis [36]. Therefore before the study we performed special work to define the accuracy of the methods used on the example of the water of lake Gorkoye (41). Thus in Table 2 all values with only significant digits and estimated analytic method errors are shown. The waters of studied lakes had a similar ratio of the main cations. Thus, sodium and magnesium were predominant cations, the Na/Mg-ratio varies in the narrow range (0.15 to 0.28) regardless of salinity. The lakes could be attributed to two types based on their anions content: chloride and chloride-sulfate ones. The waters were weak alkaline with pH values ranging from 7.5 to 9.2. The highest рН value equal to 9.2 was observed for Khoroshee lake (47) in 2009. The measured Eh values ranging from 100 to 420 V indicate that the waters of the lakes were saturated by oxygen. The lowest Eh values (100 to 170) were observed in lakes Gorkoye (42) in 2009, Presny Prud (43) in 2008 and 2009, and Gorkoye (51) in 2009. The significant variation in salinity value was observed depending on a water type. For example, Gorkoye (42) and Krugloye (45) lakes had strong brines, and their salinity levels varied in 210–290 g L^−1^. Lake Gorkoye (41) has an intermediate brine; (150 g L^−1^). While salinity value of Lake Razboynoye (46) was ten times lower, wherefore it belonged to the weak brines.

**Table 2:** The physical-chemical parameters of the waters: pH, Eh, salinity and ions content in lakes (2008 and 2009)

Water type		Year	Chloride				Chloride-sulfate						
Lake N			41	42	45	46	43	44	47	48	49	50	51
Parameter	Error												
pH	±0.05	2008	7.9	7.5	7.7	8.7	8.0	8.0	7.9	8.0	8.0	7.7	8.0
		2009	8.1	7.7	7.7	8.7	7.9	8.3	9.2	8.0	n.d.	n.d.	8.0
Eh (mV)	±20 mV	2008	360	280	n.d.	320	100	320	260	290	290	270	310
		2009	340	130	330	380	170	420	320	320	n.d.	n.d.	120
Salinity (g L^−1^)	±10 %	2008	150	290	210	12	4.3	74	240	230	230	250	230
		2009	170	280	290	16	4.3	43	99	190	n.d.	n.d.	130
Cl^−^ (g L^−1^)	±5 %	2008	80	140	110	5.6	1.2	31	96	71	84	100	82
		2009	100	170	140	7.5	0.6	25	64	96	n.d.	n.d.	52
SO_4_^2−^(g L^−1^)	±10 %	2008	19	40	30	1.5	1.2	20	65	82	66	68	80
		2009	9.9	39	30	1.9	2.1	3.6	4.5	37	n.d.	n.d.	36
NO_3_^−^(mg L^−1^)	±30 %	2008	130	380	330	31	4.0	86	330	380	380	610	460
		2009	990	1500	1500	2.2	1.8	170	730	1100	n.d.	n.d.	580
НCO_3_^−^ (mg L^−1^)	±30 %	2008	450	290	260	500	340	150	360	320	330	460	400
		2009	480	340	530	800	380	350	150	580	n.d.	n.d.	600
Na^+^ (g L^−1^)	±10 %	2008	39	8.0	59	4.0	1.1	19	6.0	63	65	63	60
		2009	37	55	79	3.8	0.9	11	24	43	n.d.	n.d.	3.0
K^+^ (mg L^−1^)	±30 %	2008	150	270	200	96	8.4	100	150	140	130	220	220
		2009	160	330	220	120	13	76	350	160	n.d.	n.d.	160
Mg^2+^ (g L^−1^)	±10 %	2008	11	20	10	1.0	0.3	4.0	11	14	14	17	11
		2009	10	13	11	0.9	0.3	2.5	4.5	11	n.d.	n.d.	5.8
Ca^2+^ (mg L^−1^)	±30 %	2008	440	300	230	32	110	350	67	390	220	250	330
		2009	613	500	630	45	98	350	1600	530	n.d.	n.d.	660
Mg/Na-ratio	-	2008	0.28	0.22	0.17	0.25	0.23	0.21	0.16	0.22	0.22	0.27	0.18
		2009	0.27	0.22	0.15	0.23	0.28	0.22	0.19	0.25	n.d.	n.d.	0.16

Most chloride-sulfate lakes had strong brines and with salinity ranging from 200 to 250 g L^−1^. These lakes resemble each other in some aspects. Lake Dolgoye (44) had a weak brine (74 g L^−1^). Presny prud (43) had the lowest salinity with salinity value equal 4.3 g L^−1^. The highest percentage of hydrocarbonate was observed for the least saline waters.

A comparison of the data obtained in 2008 and in 2009 does not show the considerable changes in the most of waters physical-chemical parameters. However, we should mention the noticeable increase of the salinity for chloride lakes in 2009, while salinity values of chloride-sulfate lakes significantly decreased. These data correlate with the increase of chloride content in the former and its decrease in the latter, as well as with the decrease of sulfate and sodium content in all the lakes, while nitrate and potassium content increased. No significant changes were observed for other parameters. We observed a noticeable difference of the water samples of 2008 and 2009 taken from three lakes. Lake Khorosheye (47) and Lake Solenoye (48) had significantly lower salinity in 2009, while the ion content was shifted from sulfate-chloride to predominantly chloride. This was especially pronounced for Lake Khoroshye (47), where salinity decreased 2.5 times and sulfate content changed from 65 g L^−1^ in 2008 to 4.5 g L^−1^ in 2009. Salinity of Presny prud (43) remained the same, while sulfate content increased two times. No changes were observed in magnesium and sodium content.

### Dominating microbial communities

During our fieldwork, we noticed that the littoral zone of some lakes contains microbial communities visible to the naked eye. The littoral zone of the lakes Gorkoye (41) and Solenoye (48) is covered by loose granular dark green and yellow-green communities with whitish and purple inclusions, which are inhabited by large numbers of *Artemia* sp. The communities from these two lakes were similar in appearance and content despite the fact that they are located far from each other and have different salinity. According to the microscopic analysis, these loose mats were aggregates of the Cyanobacteria *Aphanocapsa* sp., *Gloeocapsa* sp., and *Microcystis* sp. with inclusions of many diatom Algae.

Microscopic analysis of microbial mats found an underlayer of salt and sand in the littoral zones of the Lakes Dolgoye (44), Krugloye (45), and Khorosheye (47) demonstrating that these mats are dominated by the filamentous Cyanobacteria *Scytonema* sp.

In the littoral zone of Lake Dolgoye (44) mats of filamentous green Algae underlayed by rosy layers of purple Bacteria resembling *Thiocapsa* were found in addition to sandy mats. Characteristics of the main microbial communities are given in Table 3.

**Table 3:** Types of shoreline microbial communities observed in the studied lakes

Community type	Description	Environmental parameters	Location	Dominant species
Algae-cyano bacterial	Abundant loose dark green or yellow-greenish communities with high numbers of *Artemia* sp.	Shoreline. Salinity of 150–230 g L^−1^, рН 7.9-8.1	Lakes Gorkoye (41) and Solenoye (48)	*Aphanocapsa* sp., diatom algae
Algae-bacterial 1	Continuous or separate green patches	Littoral organic rich zone fertilized by cattle; large or small mat size	Lakes Dolgoye (44) and Razboynoye (46)	Filamentous green algae
Algae-bacterial 2	Thin easily desintegrating green layers	Littoral water-free zone, under a sand layer	Lake Krugloye (45)	Green multicellular and colonial algae
Cyano bacterial	Felted green filamenous formations	Littoral water-free zone, under layers of sand and salt	Lakes Dolgoye (44), Krugloye, (45), and Khorosheye (47)	*Scytonema* sp. filamentous cyanobacterium
Bacterial	Rosy-crimson water	Purple bacteria in little pools rich in biogenic elements under a layer of green algae	Lake Dolgoye (44)	*Thiocapsa* sp. purple bacterium

### Structure of planktonic microbial communities

The spatial and temporal organization of planktonic microbial communities of 11 lakes was studied using fluorescent in situ hybridization and microscopy. We determined total quantities of Archaea, Bacteria, Cyanobacteria, and Algae. So, for Archaea and Bacteria, populations varied from 1.7 × 10^4^ to 5.4 × 10^7^ cells mL^−1^, and from 5.9 × 10^4^ to 3.9 × 10^7^ cells mL^−1^, respectively. Algae and Cyanobacteria were from 1.0 × 10^3^ to 6.8 × 10^4^ cells mL^−1^. In general, the total quantity of microorganisms in the water samples from the lakes ranged from 1.25 × 10^5^ to 9.0 × 10^7^ cells mL^−1^.

**Fig. 2:**
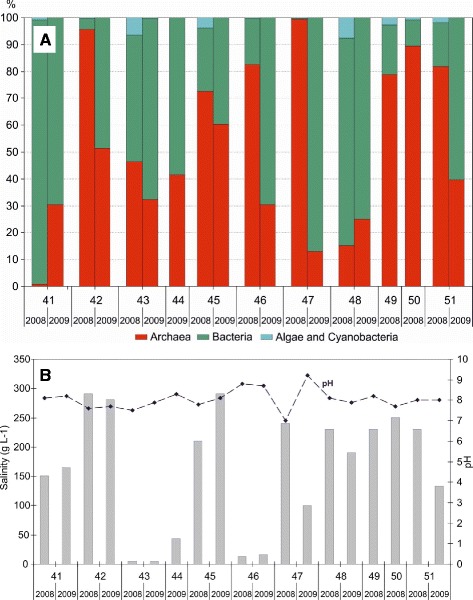
Quantities of main phylogenetic groups in plankton in the studied lakes, salinity and pH. **a** Relative abundance of Archaea, Bacteria, Algae and Cyanobacteria. **b** Salinity and pH profiles

In 2008, a maximum value for the total quantity of microorganisms (2.5 × 10^7^ cells mL^−1^) was observed in 2008 in Lake Khorosheye (47) with salinity at 240 g L^−1^. A high quantity (1.7 × 10^7^ cells mL^−1^) was also observed in Lake Gorkoye (42) at 290 g L^−1^. We should note that the high levels of microorganisms were caused by a high quantity of Archaea. Archaea represented 99.5 % of the total quantity of microorganisms (Fig. 2).

A maximum quantity of Bacteria (2.0 × 10^6^ cells mL^−1^) was observed in Lake Gorkoye (41) with salinity of 150 g L^−1^, and a high quantity of Bacteria (9.2 × 10^5^ cells mL^−1^ and 6.9 × 10^5^ cells mL^−1^) was also found with salinity of 12 g L^−1^ (Lake Razboynoye (46)) and 290 g L^−1^ (Lake Gorkoye (42)), respectively. The quantity of Algae and Cyanobacteria (from 8.0 × 10^3^ to 5.9 × 10^4^ cells mL^−1^) was generally an order of magnitude lower than Bacteria. Relatively high percentage of Algae and Cyanobacterial was observed in Lake Presny prud (43), Lake Krugloye (45), and Lake Solenoye (48) (Fig. 2). The highest abundance (in absolute value) of algae and cyanobacteria was observed in lakes Gorkoye (42) - 3.6 × 10^4^ cells mL^−1^, Khorosheye (47)-3.8 × 10^4^ cells mL^−1^, and Gorkoye (51)-5.9 × 10^4^ cells mL^−1^, and were mainly caused by an extensive growth of halophylic Algae (*Dunaliella* sp.) that were detected using microscopy.

On the whole, Archaea dominated in 7 of the 10 samples in 2008, making up over 70 % of the total cell number. In two cases Bacteria dominated, making up 98 % and 77 % of the cells. The percentage of Algae and Cyanobacteria was only 0.2 and 7.7 %, respectively (Fig. 2). However, they made a high contribution to the biomass production and ecosystem functioning due to their high biomass. For example, in Lake Gorkoye (42), an average size of an algal cell was 500 μm^3^, while for an average bacterial cell it was only 5 μm^3^, and for an average archaeal cell, it was less than 1 μm^3^. We should also note that Archaea formed mucous microcolonies with small indiscernible cells in these communities.

In some cases we observed microcolonies of small bacterial and archaeal cells, which grew together, and areas where bacterial and archaeal probes overlapped.

In 2009, the maximum quantity of Archaea (3.6 × 10^7^ cells mL^−1^) was detected in Lake Krugloye (45) along with the highest salinity, 290 g L^−1^. One of the highest bacterial densities (6.8 × 10^4^ cells mL^−1^) was also observed in this lake. In 2009, the decrease of salinity was accompanied by a decrease in all groups of microorganisms (Fig. 2). The only exception was in Lake Dolgoye (44), where high quantities of Archaea (2.8 × 10^7^ cells mL^−1^), Bacteria (3.9 × 10^7^ cells mL^−1^), Algae, and Cyanobacteria (3.0 × 10^4^ cells mL^−1^) were observed at low salinity levels (43 g L^−1^). The adjacent lakes Dolgoye (44) and Krugloye (45), which differ significantly in physical-chemical and microbiological parameters, stress the unique nature of the lakes studied in the region.

On the whole, the proportion of Bacteria was higher in 2009. Archaea dominated only in one sample with a high salinity (Fig. 2). The percentage of algal and cyanobacterial cells was negligible.

Of the total 19 water samples (2008 and 2009), Archaea and Bacteria dominated in nine communities, the former ones usually dominated at higher salinity values (Fig. 2). The maximum percentage of Archaea (99.5) was observed at the salinity level of 280–290 g L^−1^, while a high percentage of Bacteria (98.4) was found at lower salinity values, 4 to 190 g L^−1^. Algae and Cyanobacteria never dominated any planktonic communities. A rather high percentage of Algae and Cyanobacteria (7.7 %) was found in Lake Solenoye (48), due to a considerable proportion of *Aphanocapsa* sp., *Gloeocapsa* sp., and *Microcystis* sp. colonial Cyanobacteria, as well as in Presny Prud (43) (6.4 %), which harboured many green protococcous Algae. In all other cases it did not exceed 4 %, and was usually below 1 %.

### Correlation of plankton abundance and environmental factors

The physical-chemical parameters of water from the lakes and the data on the number of Archaea, Bacteria, Algae and Cyanobacteria, and the total number of microorganisms were used as the initial data. Abundance and environmental parameters make high contributions to both components, which indicate that there is a relationship between these variables.

Three principal components were selected for analysis. Together they explain 71.1 % of the observed variation. The first principal component explains 37.3 % of the observed variability. The salinity (*r* = 0.99) and Cl, SO_4_^2−^, NO_3_^−^, Na^+^, К^+^, Mg^2+^ ion contents (*r* = 0.64–0.97) have positive correlation with this component. pH was in antiphase (*r* = −0.62) (Table 4).

**Table 4:** Correlation coefficients with their significances between the principal components and bacterial and environmental parameters

Parameter	Principal components					
	PC1		PC2		PC3	
	Coeff.	*p*	Coeff.	*p*	Coeff.	*p*
рН	−0.6203	0.0037	0.3054	0.2071	0.4552	0.0494
Eh	−0.0233	0.9257	0.2626	0.2821	−0.0251	0.9200
Cl^−^	0.9375	<0.0001	0.1491	0.5481	0.1063	0.6695
SO_4_^2−^	0.7253	0.0002	−0.4454	0.0554	−0.0885	0.7225
NO_3_^−^	0.6404	0.0024	0.5211	0.0208	0.2703	0.2676
НCO_3_^−^	−0.2834	0.2438	0.3284	0.1725	−0.3730	0.1170
Na^+^	0.9676	<0.0001	−0.1339	0.5901	−0.0656	0.7927
K^+^	0.6996	0.0005	0.3325	0.1668	0.4687	0.0420
Mg^2+^	0.9016	<0.0001	−0.2295	0.3499	0.0348	0.8892
Ca^2+^	0.1807	0.4648	0.4641	0.0444	0.8025	<0.0001
Mg/Na	−0.4240	0.0703	−0.1999	0.4176	0.0644	0.7963
Salinity	0.9894	<0.0001	−0.0647	0.7956	0.0058	0.9814
LgA	0.2673	0.2731	0.6482	0.0020	−0.5615	0.0111
LgB	−0.1333	0.5916	0.8897	<0.0001	−0.1066	0.6688
LgAlg	0.3624	0.1288	−0.1809	0.4644	−0.6440	0.0022
LgS	0.1795	0.4680	0.8262	0.0000	−0.4595	0.0470

For each parameter were calculated the correlation coefficient (Coeff.) and statistical significance (p); LgA-logarithm of the number of Archaea, LgB-logarithm of the number of Bacteria, LgAlg-logarithm of the number of Algae and Cyanobacteria, LgS-logarithm of the total population

The second component explained 19.6 % of the observed variability. The logarithm of the number of Bacteria (*r* = 0.89), the logarithm of the total population (*r* = 0.83), the logarithm of the number of Archaea (*r* = 0.65) and the content of NO_3_^−^(*r* = 0.52) mainly contribute to this component (Table. 4). The third component explained 14.2 % of the observed variability. It had a positive correlation with the content of Ca^2+^ (*r* = 0.80) and a negative correlation with the logarithms of the number of Algae and Cyanobacteria (*r* = −0.64), Archaea (*r* = −0.56) and total population (*r* = −0.46) (Table 4).

Interannual specificity was shown by the second principal component. An exception was the freshwater lake Presny Prud (43) (2009), located in the area studied in 2008. Perhaps the reason for this was a very low content of nitrates in the water in 2009, which was closer to the average content of nitrates in the water of the lakes in 2008 than it was to the average content of nitrates in the water of the lakes in 2009.

The greatest differences in the third principal component were detected in Lake Khorosheye (47), possibly, due to the higher order of Ca^2+^ in the water in 2009.

Therefore, to identify a clear relationship between the biological and physicochemical components of the ecosystem, we used the 2B-PLS method. Analysis by the 2B-PLS method showed that 96.2 % of the total covariance are accounted for by the first two datasets, or blocks. 59.6 % were accounted for by the first couple of datasets, 36.6 %-for the second pair, and less than 4 % of the covariance were accounted for by the remaining 4 pairs of datasets. For the first pair of datasets, the correlation coefficient was 0.5 (*P* = 0.029), for the second pair 0.7 (*P* = 0.00085).

**Fig. 3:**
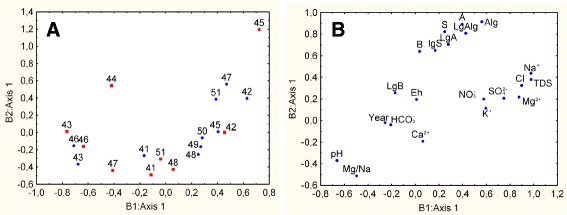
Results of the data analysis by the method 2B-PLS on first pair of datasets. Results of the data analysis on the quantity of different groups of microorganisms and factors potentially affecting. The B1: Axis 1-salinity datasets, the B2: Axis 1-datasets of microbial abundance. **a** Blue circles-2008; red squares-2009; 41–51-lakes. **b** Symbols (hereinafter): LgA-logarithm of the number of Archaea, LgB-logarithm of the number of Bacteria, LgAlg-logarithm of the number of Algae and Cyanobacteria, LgS-logarithm of the total population; A-number of Archaea, B-number of Bacteria, Alg-number of Algae and Cyanobacteria, S-total number of microorganisms, TDS-salinity

**Fig. 4:**
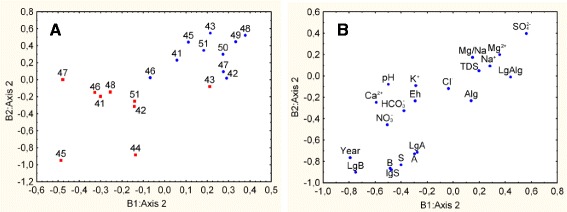
Results of the data analysis by the method 2B-PLS on second pair of datasets. Results of the data analysis on the quantity of different groups of microorganisms and factors potentially affecting. The B1: Axis 2-salinity datasets, the B2: Axis 2–datasets of abundance. **a** Blue circles-2008; red squares-2009; 41–51-lakes. **b** Symbols (hereinafter): LgA-logarithm of the number of Archaea, LgB-logarithm of the number of Bacteria, LgAlg-logarithm of the number of Algae and Cyanobacteria, LgS-logarithm of the total population; A-number of Archaea, B-number of Bacteria, Alg-number of Algae and Cyanobacteria, S-total number of microorganisms, TDS-salinity

B1 is a salinity (concentration of main ions) datasets, B2 is datasets of microbial abundance (Figs. 3, 4). Lakes with high salinity and relatively high abundance of Algae and Cyanobacteria represent one group of lakes. Another group of lakes was characterized by a relatively high pH, high abundance of Archaea, Bacteria, a total number and a certain ratio of Mg/Na (Figs. 3a, 4a).

A second two datasets, or blocks clearly showed an interannual aspect (Fig. 4). It is evident that the lakes in 2008 had a high content of sulfates, high salinity and abundance of Algae and Cyanobacteria, and in 2009 the number of Bacteria, Archaea, and total quantity was greater. Minimum distances characterizing the differences in the first pair datasets were intrinsic to the lakes Presny prud (43), Razboynoye (46), Gorkoye (41) and Solenoye (48), the maximum distances–to the Krugloye (45) and Khorosheye (47) lakes. Minimum distances characterizing the differences on the second pair of datasets were intrinsic to the lakes Presny prud (43) and Razboynoye (46), the maximum distances-to the lakes Krugloye (45), Khorosheye (47), and Solenoye (48).

In general, the greatest interannual changes were intrinsic of the lakes Krugloye (45) and Khorosheye (47), possibly due to significant fluctuations in salinity and other physical-chemical parameters as well as to an abrupt change in the quantity of Bacteria and Archaea over different years. The lowest interannual changes were intrinsic to the lakes with more freshwater, Presny prud (43) and Razboynoye (46). Significant fluctuations in the quantity were also been observed for these lakes, while physical-chemical parameters were more stable.

The application of the 2B-PLS method in this case was completely acceptable because the dependency on salinity and abundance of microorganisms was observed in the first two datasets. And the dependence on the clear interannual variability was observed in the second two pairs of datasets.

## Discussion

The results of our study demonstrate that microbial communities of saline lakes of the Novosibirsk region have considerable microbial diversity. The distribution of the different groups (Bacteria, Archaea, Algae, and Cyanobacteria) in the lakes was highly heterogeneous. So, for Archaea and Bacteria, oscillations of populations were 3 orders of magnitude greater. Archaea dominated in 9 of the 19 populations. Algae and Cyanobacteria were the lowest and the most consistent in quantity. The total cell frequencies and frequencies of the main phylogenetic groups are similar to those of other water ecosystems. Thus, in the chemocline of Lake Shunet which has the salinity levels of 17–66 g L^−1^, FISH-TSA revealed 10^7^ cells mL^−1^ of the total microbial numbers, the numbers of Bacteria and Archaea and in the chemocline of Lake Shira which has the salinity levels of 14–18 g L^−1^, FISH-TSA revealed 10^7^ cells mL^−1^ of the total microbial numbers and 10^6^ cells mL^−1^ per the numbers of Bacteria and Archaea [11, 47, 48].

The water from the continental slope of the Black Sea contained 10^5^ cells mL^−1^ of sulfate reducing Bacteria as revealed by FISH, and 10^6^, by DAPI [13]. The delta of the Selenga river harboured 10^6^ cells mL^−1^ as was demonstrated by DAPI [12]. There is no published information on microbial communities for the lakes in our region, but there are data on the numbers of Anoxyphotobacteria that could reach 10^8^ cells mL^−1^ on selective media [35]. Our results do not conform to other studies of freshwater and soil microbial communities, which report lower numbers of cells detected by hybridization with bacterial and archaeal probes than those visualized by DAPI staining [49]. However, in the case of highly saline ecosystems the abundance of archaeal-bacterial microcolony can be explained by protective mechanisms which include mutualistic growth and the synthesis of a common mucous matrix. The location of Bacteria and Archaea in such microcolony may be a sign of close interaction among these groups and an indicator of common metabolic pathways [3].

Filamentous Algae and colonial and filamentous Cyanobacteria dominated in the studied shoreline communities, which is characteristic for all saline lakes. However, we should note that lakes of the Novosibirsk region are unique in the prevalence of Cyanobacteria of the genera *Scytonema*, and *Aphanocapsa*, while in other regions, *Microcoleus chthonoplastes*, *Halospirulina tapeticola*, and *Aphanothece halophytica* [50], and *Phormidium* or *Oscillatoria* [51] are usually dominant.

The obtained data on the physical-chemical parameters of water of eleven saline lakes of the Novosibirsk region allowed us to estimate temporal changes in different lakes. We found the considerable changes in salinity and some other parameters, which is characteristic of small lakes in cryoarid regions [52].

The data on physical-chemical parameters and the quantities of Bacteria, Archaea, Algae, and Cyanobacteria allowed us to employ statistical analyses to look for patterns of organism distribution.

Some recent studies showed that environment is crucial in the formation of planktonic microbial communities, and that it is predominantly the local environment that regulates community composition [41, 43, 53, 54]. For ninety-eight shallow lakes located in three European regions there is strong evidence that species sorting in response to local environmental factors is a key determinant of the taxon composition of aquatic bacterial communities over a very broad range of spatial scales. The main factors controlling bacterial community composition were resources (TN, total N) and grazing-related factors (e.g., zooplankton biomass) [41]. For six high altitude lakes in the Mount Everest region (Nepal) community composition depended mostly on distance between these lakes, but certain local environmental factors were also significant in explaining this apparent biogeographic pattern [43]. Multiple stepwise regression analysis for the Zapadłe lake in North-Eastern Poland showed the following factors determined prokaryotic abundance: conductivity, TN, depth, temperature, Fe, DOC and N_org_ (*r*^2^ = 0.89, *P* < 0.05), while conductivity, TOC, H_2_S and PO_4_ influenced prokaryotic biomass at *r*^2^ = 0.92 (*P* < 0.05). Canonical correspondence analysis (CCA) indicated that conductivity, H_2_S, DON, TOC, DOC, N_tot_, N_org_, NH_4_, P_tot_, P_org_, PO_4_, and Mn explained changes in bacterial community structures in June and September, while the most important measured factor in November was temperature [53]. For the intertidal coast of the Río de la Plata estuary (Argentina) were found that the plankton community assemblage was significantly modified by high eutrophication levels along the intertidal southwest coast of the estuary [54]. The authors observed that high densities and biomasses of bacterioplankton, picophytoplankton (chlorophytes and cyanobacteria), and microzooplankton (rotifers, aloricate ciliates, and tintinnids) were positively related to highly eutrophic and polluted sites along the inner portion of the Río de la Plata estuary. In contrast, the biomasses of the microphytoplankton (diatoms) and mesozooplankton (copepods) were positively correlated with the high turbidity and conductivity levels in the estuary’s outer and less polluted area. The biomass of picophytoplankton (chlorophytes chlorococcales, cyanobacteria) and nanophytoplankton (small diatoms) was positively associated with the most polluted area characterized by high concentrations of P-PO_4_^−3^ and N-NH_4_^+^ and the greatest bacterial density and biomass [54].

For small lakes on the shore of the Baltic Sea, three variables (salinity, chlorophyll a concentration, and water color) were found to have great impact on the composition of bacterial communities. There is a strong aspect of environmental determinism shaping aquatic microbial assemblages on a global scale, and environmental forces are acting singularly on different bacterial groups [42]. For two shallow alkaline pools in Eastern Austria bacterial and cyanobacterial numbers depend on abiotic parameters, such as wind, precipitation, solar radiation, and temperature [39]. Impact of temperatures was confirmed for a soda lake in Transbaikal region [44]. For five shallow soda lakes in Eastern Austria agitation, salinity, conductivity, and dissolved organic carbon were found to have the biggest impact on Bacteria and Cyanobacteria [40]. For saline lakes of Mongolia, China, and Argentina, bacterial distribution was found to depend on environmental (ion concentration and pH) and spatial factors [46]. And for the Sfax saltern (Tunisia) it was found that the salinity gradient had a considerable influence on the composition of the phytoplanktonic communities [45].

Therefore, there is a set of environmental factors, e.g. salinity, chlorophyll a concentration, water color, wind, precipitation, solar radiation, temperature, conductivity, and dissolved organic carbon, etc., that has an impact on microbial communities; relative impact of a factor depends on ecosystem type and location [39, 40, 42, 44–46]. However, in the case of our study we acknowledge that the first stage of our statistical analysis, the PCA method is not a good method for identifying the relationship between the abundance of microorganisms and the environmental parameters in saline lakes of the Novosibirsk region, because it is designed for describing sets of uniform characters and objects as it was made in [55].

Therefore, at the second stage of analysis we used the 2B-PLS method, which allowed us to suggest that the structure of microbial communities in the studied lakes is determined by physical-chemical parameters, most significantly by salinity (concentration of main ions). Thus, increase of salinity in microbial communities results in the increase of archaeal percentage, while decrease leads to increase in bacterial abundance. Generally changes in salinity resulted in changes of total plankton quantity, as well as in the ratios of the main phylogenetic groups: Archaea, Bacteria, Algae, and Cyanobacteria. In the majority of cases the increase in absolute and relative quantities of Archaea was observed. Salinity is a factor that determines ionic strength of solutions. Microorganisms can tolerate a wide range of salinity. Changes in salinity level are compensated by ion pumps and Na-H^+^ antiporters. These changes of internal solute potential require a significant amount of energy. As a result, a cell growing at a non-optimal modulator level may be at a competitive disadvantage in a given community. This may result in community change [56].

## Conclusion

We detected high microbial richness in the communities of saline lakes of the Novosibirsk region, and found the dominant species in the plankton and benthos communities. Filamentous and colonial Cyanobacteria of the genera *Scytonema*, *Aphanocapsa*, and/or filamentous Algae dominated in littoral communities. Spatial and temporal organization of planktonic microbial communities and the quantities of Archaea, Bacteria, Algae, and Cyanobacteria were investigated using fluorescent in situ hybridization and microscopy. We have found that the dominant planktonic component is represented by Archaea, or, less frequently, by Bacteria.

Studies of the impact of environmental factors on microbial communities of saline lakes of the Novosibirsk region allowed us to detect statistically significant relationships between the quantities of organisms belonging to various phylogenetic groups and physical-chemical parameters.

We found that salinity (concentration of main ions) is the main but not the only factor affecting community structure. Generally changes in salinity resulted in changes of total plankton quantity, as well as in the ratios of the main phylogenetic groups: Archaea, Bacteria, Algae, and Cyanobacteria. In the majority of cases the increase in absolute and relative quantities of Archaea was observed.

Our study demonstrates that saline lakes in the Novosibirsk region contain the unique microbial communities that may become a prolific source of microorganisms for fundamental and applied studies in various fields of ecology, microbiology, geochemistry, and biotechnology, and deserve further metagenomic investigation.

## Methods

### Object of the study

During two expeditions in June 2008 and in July 2009 we studied 11 saline lakes located in the south of the Novosibirsk region (Table 1, Fig 1). All of them are shallow, less than 4 m deep; some are intermittent. Since many lakes in this region have same names, we gave numbers to each sampled lake (Fig. 1). All but two (№ 42 and 43 lakes are located within an Upper Pleistocene-Holocene lakes, that was probably similar to the modern Chany lakes, which has salinity ranging from 0.75 to 6 g L^−1^ [57, 58].

Samples of water and microbial mats were collected in littoral zones of the lakes into sterile tubes and containers which were left either unfixed or fixed using ethanol in final concentration 50 % (samples of water) or 4 % paraformaldehyde (samples of microbial mats and water). Samples were mixed with 3 volumes of 4 % paraformaldehyde and kept at 4 °C for 3–13 h [59]. Over 200 samples were taken for microbiological analyses. Over 100 samples were analyzed by the FISH method. Water samples were analyzed using FISH and microscopy. Samples of microbial mats were analyzed using microscopy. Each sample was selected and analyzed in triplicate.

### Water parameters

Salt-lake solutions were collected during the field investigations in July 2008 and June 2009. Samples were taken in the littoral zone, directly in the habitat being studied microbial communities. Three parallel samples were collected using plastic containers (0.5 L in volume). The solutions were filtered through a sterile membrane filter with a pore diameter of 0.45 μm. Two of the samples were stabilized by concentrated HNO_3_ in the amount 2 mL per 0.5 L of solution for determination the metal contents. pH and Eh were determined on site using portable field instrument kit Ob (Russia). A water analyzer ANION 7051 (Russia) and multichannel combined analyzer ANION 4151 (Russia) were used for measurements. The pH was measured using a combined pH electrode ESLK-01.7 (Russia). The Eh was registered by measurement of voltage between platinum electrode and clorine-silver reference electrode. A standard solution of potassium ferrocyanid and potassium ferricyanid was used for calibration.

The concentrations of nitrates and chlorides in the lake surface solutions, pore solutions of the bottom sediments, and the solution of the microbial community were measured in a potentiometric way using an ANION 4151 multichannel combined analyzer, ion-selective electrodes Ekom-NO_3_, Ekom-Cl (Russia) and clorine-silver reference electrode.

The contents of inorganic and organic carbon in the solutions were registered with a Total Organic Carbon Analyzer, TOC-VCSH (Shumadzu, Japan). НCO_3_^−^-content was calculated from the content of organic carbon

The content of main cations (Na, K, Mg, Ca) and sulfur was determined by inductively coupled plasma optical emission spectrometry (ICP-OES) with a Optima 4300 DV (PerkinElmer Inc., USA). The content of SO_4_^2−^ anion was calculated from amount of sulfur determined by ICP-OES. The control of the cations (Na, Mg, K, Ca) concentrations in the water samples was also carried out by AAS (Thermo Electron Corporation Solar M6 spectrometer), with measured concentrations from 0.05 to 5 · 10^5^ ppm.

The value of salinity was calculated as a sum of concentrations for the all cations and all anions using the above described analytical data.

### Fluorescent in situ hybridization

FISH was performed as it was described earlier [3]. The following standard probes were used: for Bacteria, EUB338 (5′-GCTGCCTCCCGTAGGAG-3′ [60]; for Archaea, ARCH915 (5′-GTGCTCCCCCGCCAATTC-3′ [5]. To increase signal intensity we also employed additional probes constructed using the Vector NTI program: for Bacteria, EUB1097 (5′-GGGTTGCGCTCGTTRCG-3′); for Archaea, ARCH1195 (5′-TTCGGGGCATrCkGACCT-3′). Probes labelled with CY-5 and FAM were synthesized by LLC “Medigen Laboratory” (Novosibirsk).

Fixed cells were concentrated 10–20 times by centrifugation at 4000 g for 10 min. The supernatant was discarded, precipitate was washed with 1.5 ml of 50 % ethanol to remove residual salt followed by centrifugation at 4000 g for 10 min for a total of three times. Cells were then resuspened in 50 % ethanol. About 20 μL of the cells were transferred on to slides containing 1 % fixed gelatine, dried for 15 min at 46 °C. A total of 20 μL of cell suspension was immobilized on to slides with fixed 1 % gelatine, dried at 46 °C for 15 min, and dehydrated for 3 min in 50 %, 80 %, and 96 % ethanol. Slides were dried at 46 °C for 10 min and stored at room temperature.

For hybridization, 30 μL of solution containing 0.9 M NaCl, 20 mM Tris–HCl, pН 7.3, 0.1 % SDS, and 50 ng of each probe was transferred to dehydrated slides. Reactions were performed with four probes simultaneously. The optimum formamide concentration of each probe in the process of hybridization and the way of attaining such an optimum formamide concentrations have been determined (data are not shown). Formamide concentration in hybridization buffers ranged from 0 to 60 % according to the procedure described by [59]. A slide incubated in RNAse solution (100 μg μL^−1^) at 37 °C prior to hybridization was used as a negative control [61]. Hybridization was performed in a moist chamber at 46 °C for 2 h.

After that, the slides were incubated for 20 min at 48 °C in washing buffer (0.9 M NaCl, 20 mM Tris–HCl, pН 7.3), washed in ice-cold water, dried by air flow, and stored at 4 °C in darkness.

The total number of microorganisms were counted using DAPI [62]. After hybridization, slides were stained with DAPI (8 μg mL^−1^) and then mounted with DABCO anti-fade solution (Sigma Aldrich, USA) covered by a cover slip and examined under a microscope with the × 100 ES Plan-NEOFLUAR objective.

### Microscopy

Quantity and morphological diversity of microorganisms (Archaea, Bacteria, Cyanobacteria, and Algae) were studied using light and luminescent Carl Zeiss microscopes (Axioskop 2 Plus and Axioskop А1, Germany) in the SB RAS Microscopy Center (Novosibirsk). The cells were counted at 1,000 magnification. DAPI-stained cells and the cells hybridized with probes were counted using filter sets 02 (G365, FT 395, LP420), 10 (BP 450–490, FT 510, BP 515–565) and 50 (BP 640/30, FT 660, BP 690/50) (Carl Zeiss, Germany). Photos were taken using the AxioCam 1Cc 3 camera and processed using the AxioVision Rel. 4.8 program.

The number of cells (N) per 1 mL was calculated according to the following formula: *N* = (a × S : (s × V)) × n, where a is the average number of cells in the microscopic field (diameter of the microscopic viewing field was 130 μm), S is the smear area (mm^2^), V is the volume of suspension on the slide (mL), and n is the dilution factor. Cell volumes were calculated via simple geometric approximations. For the morphometric analysis, 100 bacterial cells from each sub-sample were sized according to length and diameter.

At least 20–30 microscopic fields were examined. Since archaeal cells were very small and were observed only as microcolonies, the archaeal cell number was estimated by calculating the area of the microcolony and assuming that an archaeal cell has an area of 1 μm^2^. The same calculations were performed for small bacterial cells forming microcolonies. If archaeal and bacterial cells formed microcolonies, the areas of hybridization with archaeal and bacterial probes were calculated separately. Algae and Cyanobacteria were identified using morphological characters that were visualized by autofluorescence. Cyanobacteria were identified using the identification guides of Komárek and Anagnostidis [63, 64]. The percentage of archaeal, bacterial, and algal cells was calculated relative to the total number of microorganisms; Algae and Cyanobacteria were counted together. 

### Statistical analysis

The principal components analysis (PCA) and two-block partial least squares (2B-PLS) [65] were used to determine the main environmental factors controlling the structure of microbial communities. Calculations were performed using Statistica 8, Excel, and PAST [66].

In our study, we employed PCA as the first step of analysis, to identify the correlation between the quantity (abundance) of microorganisms and physical-chemical parameters. This method has been successfully applied to studying variation of physical-chemical parameters on the structure of microbial communities of some ecosystems [40, 44]. However, we acknowledge that the PCA method is not a good one for identifying the relationship between the abundance of microorganisms and the environmental parameters. This is not surprising, as it was intended to describe a group of homogeneous characteristics and homogeneous objects [55]. Therefore, we used the 2B-PLS method at the final stage of the analysis.
